# Guidance in author instructions of hematology and oncology journals: A cross sectional and longitudinal study

**DOI:** 10.1371/journal.pone.0176489

**Published:** 2017-04-28

**Authors:** Ingrid Toews, Nadine Binder, Robert F. Wolff, Guenes Toprak, Erik von Elm, Joerg J. Meerpohl

**Affiliations:** 1 Cochrane Germany, Medical Center - University of Freiburg, Faculty of Medicine, University of Freiburg, Freiburg, Germany; 2 Institute for Medical Biometry and Statistics, Faculty of Medicine and Medical Center - University of Freiburg, Freiburg, Germany; 3 Kleijnen Systematic Reviews Ltd, Unit 6, Escrick Business Park, Escrick, York, United Kingdom; 4 Cochrane Switzerland, Institute of Social and Preventive Medicine (IUMSP), Lausanne University Hospital, Lausanne, Switzerland; 5 Centre de Recherche Épidémiologie et Statistique Sorbonne Paris Cité – U1153, Inserm / Université Paris Descartes, Cochrane France, Hôpital Hôtel-Dieu, Paris, France; Johns Hopkins University Bloomberg School of Public Health, UNITED STATES

## Abstract

**Background:**

The debate about the value of biomedical publications led to recommendations for improving reporting quality. It is unclear to what extent these recommendations have been endorsed by journals. We analyzed whether specific recommendations were included in author instructions, which journal characteristics were associated with their endorsement, how endorsement of the domains changed and whether endorsement was associated with change of impact factor between 2010 and 2015.

**Methods:**

We considered two study samples consisting of “Hematology” and “Oncology” journals of the Journal Citation Report 2008 and 2014, respectively. We extracted information regarding endorsement of the (1) recommendations of the International Committee of Medical Journal Editors, of (2) reporting guidelines, (3) requirement for trial registration and (4) disclosure of conflicts of interest. Data extraction was done by reading the author instructions before conducting a text search with keywords. We calculated a global generalized linear mixed effects model for endorsement of each of the four domains followed by separate multivariable logistic regression models and a longitudinal analysis. We defined endorsement as the author instructions saying that they approve the use of the recommendations.

**Results:**

In 2015, the ICMJE recommendations were mentioned in author instructions of 156 journals (67.5%). CONSORT was referred to by 77 journals (33.3%); MOOSE, PRISMA, STARD and STROBE were referred to by less than 15% of journals. There were 99 journals (42.9%) that recommended or required trial registration, 211 (91.3%) required authors to disclose conflicts of interest. Journal impact factor, journal start year and geographical region were positively associated with endorsement of any of the four domains. The overall endorsement of all domains increased between 2010 and 2015. The endorsement of any domain in 2010 seemed to be associated with an increased impact factor in 2014.

**Conclusion:**

Hematology and oncology journals endorse major recommendations to various degrees. Endorsement is increasing slowly over time and might be positively associated with the journals’ impact factor.

## Introduction

The successful translation of findings from research into clinical practice depends on the timely, accurate and complete reporting of study methodology and results [1]. Still, the overall quality of study reports is moderate to poor, as several studies have shown [2–5]. Additional measures of good publication practice, such as disclosure of potential conflicts of interest, ensure that users can assess the validity of research results and apply them correctly [6, 7]. In addition, the value of biomedical research is increased by transparent and targeted research priorities. Transparent study planning, conduct and reporting are measures to reduce research waste as laid out by the Lancet series in 2014 [8, 9]. Journals’ author instructions might have potential to increase the value and transparency in biomedical research.

In the past, several recommendations have been proposed to improve reporting quality and publication practice: First, the International Committee of Medical Journal Editors (ICMJE) publishes the **“**Recommendations for the Conduct, Reporting, Editing and Publication of Scholarly Work in Medical Journals**”** [10]. This consensus document covers themes ranging from ethical aspects, such as the role and responsibilities regarding the publication of biomedical research, publication issues such as duplicate and overlapping publications, to preparation, structure and submission of manuscripts including reference style and formatting directions. To date, it has been endorsed by over 2,600 biomedical journals worldwide (http://www.icmje.org/journals.html).

Second, specific reporting guidelines, such as the CONSORT Statement for randomized parallel-group trials, were developed to help authors improve the completeness and accuracy of publications about clinical research [11]. Although it has been demonstrated in systematic reviews that there might be a beneficial effect of adhering to reporting guidelines, it has to be noted that some studies included in this review also showed a negative or no effect [12]. Whether reporting guidelines are being endorsed by biomedical journals has been studied repeatedly for general medicine [13–18] but less so for medical specialties [19–25] and, to our knowledge, not in journals of hematology & oncology.

Third, registration of clinical trials prior to patient enrollment has been advocated for years as an important step to overcome selective reporting of trial results [26]. Registration rates of hematology trials were reported to increase over time [27] and issues of selective reporting of both entire studies and individual study outcomes have been widely recognized [28].

Finally, there is a continued debate about the disclosure of potential conflicts of interest [29–32]. Authors as well as journal editors and reviewers potentially have financial ties or academic or personal interests in conflict with a manuscript that is to be accepted or rejected for publication.

We hypothesized that specific journal characteristics are associated with the endorsement of these four domains in different ways. Firstly, high profile journals pay more attention to the quality of the articles they publish and might therefore be more likely to endorse recommendations for good publication practice. Secondly, the journals’ start year might determine how established the journal is and how large its readership is. The number of readers of new journals might be limited once the journal is newly published and increase with time. Long-standing journals might have more resources for and experience in drafting, implementing and monitoring their author instructions and editorial procedures and might therefore be more likely to endorse reporting guidelines and other policies. Thirdly, editorial practices and policies may vary by geographical region of publication. Further, geographical region of publication in connection to publication language can limit the journals’ readership, for example, journals published in languages other than English have a smaller potential readership. This might have an impact on the journals’ popularity which in turn might affect the size of the editorial team and the resources available to endorse recommendations for good publication practice in author instructions. Additionally, the limited availability of reporting guidelines in languages other than English might pose a barrier to their endorsement and adherence.

### Study objectives

Our first objective was to elucidate whether journals’ author instructions endorse recommendations or requirements on adherence to selected recommendations from four domains for transparent reporting. In the second objective, we sought to answer whether journal characteristics are associated with the endorsement of the domains. The third objective was to detect time trends in the endorsement of the four domains between 2010 and 2015. Our fourth objective was to analyze whether the endorsement of any domain was associated with changes of the journal impact factor between 2010 and 2014.

## Methods

We used a cross-sectional study design to examine the current author instructions in “hematology” and “oncology” journals. In addition, we employed a longitudinal design to examine the development of the content of the author instructions in these journals over time.

### Study sample

**Fig 1:**
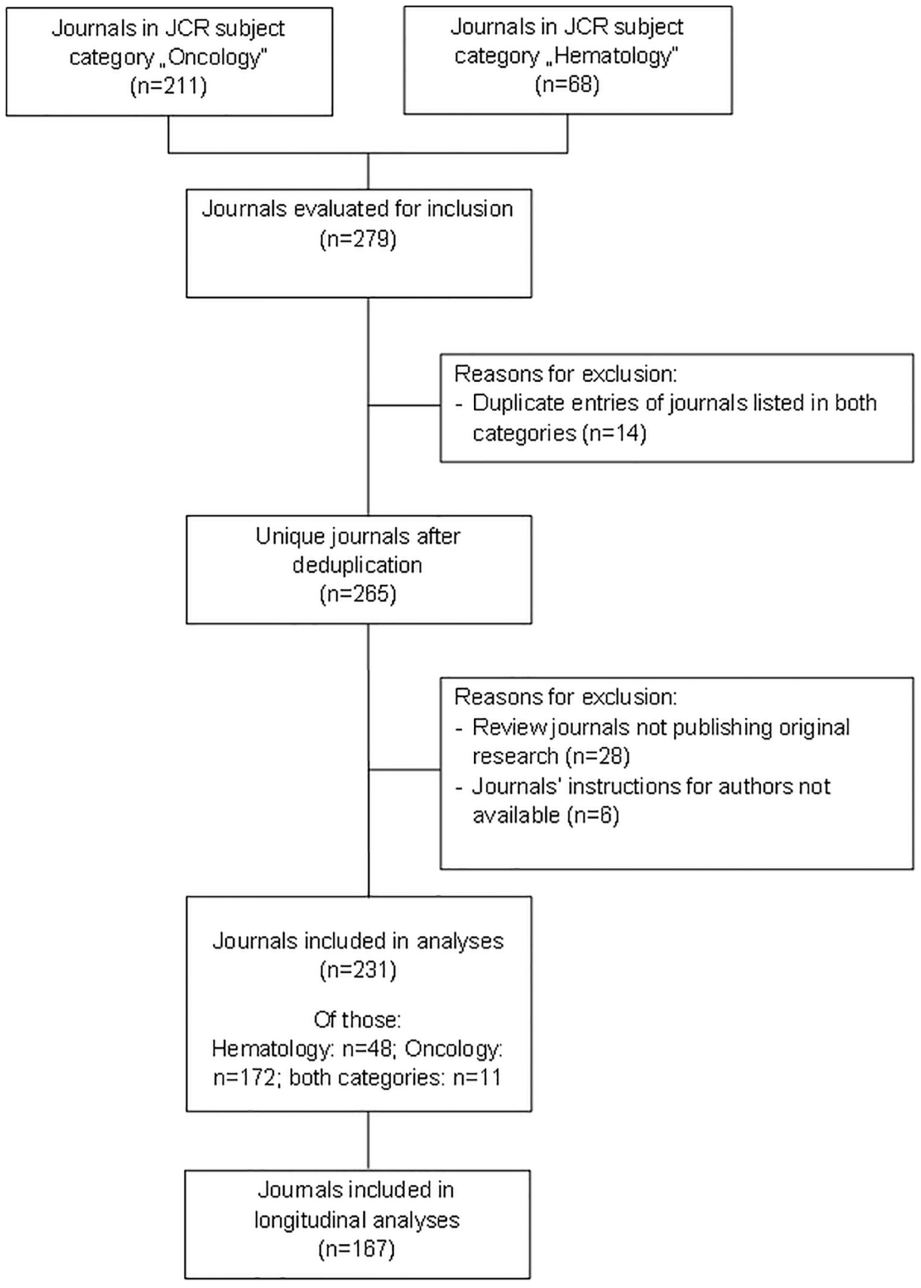
Flow chart—Journal selection for the cross sectional study part.

For the cross-sectional part of the study, we accessed the most recent Journal Citation Report (JCR 2014) (Science Edition [33]) through the Institute for Scientific Information’s Web of Knowledge web site and compiled a list of all journals in the subject categories “Hematology”and “Oncology”in July and August 2015 (Fig 1). Duplicate entries were removed in case they were listed in both categories. Furthermore, journals which did not report on original research in their published articles and whose author instructions were not available online for analyses were excluded (Fig 1).

**Fig 2:**
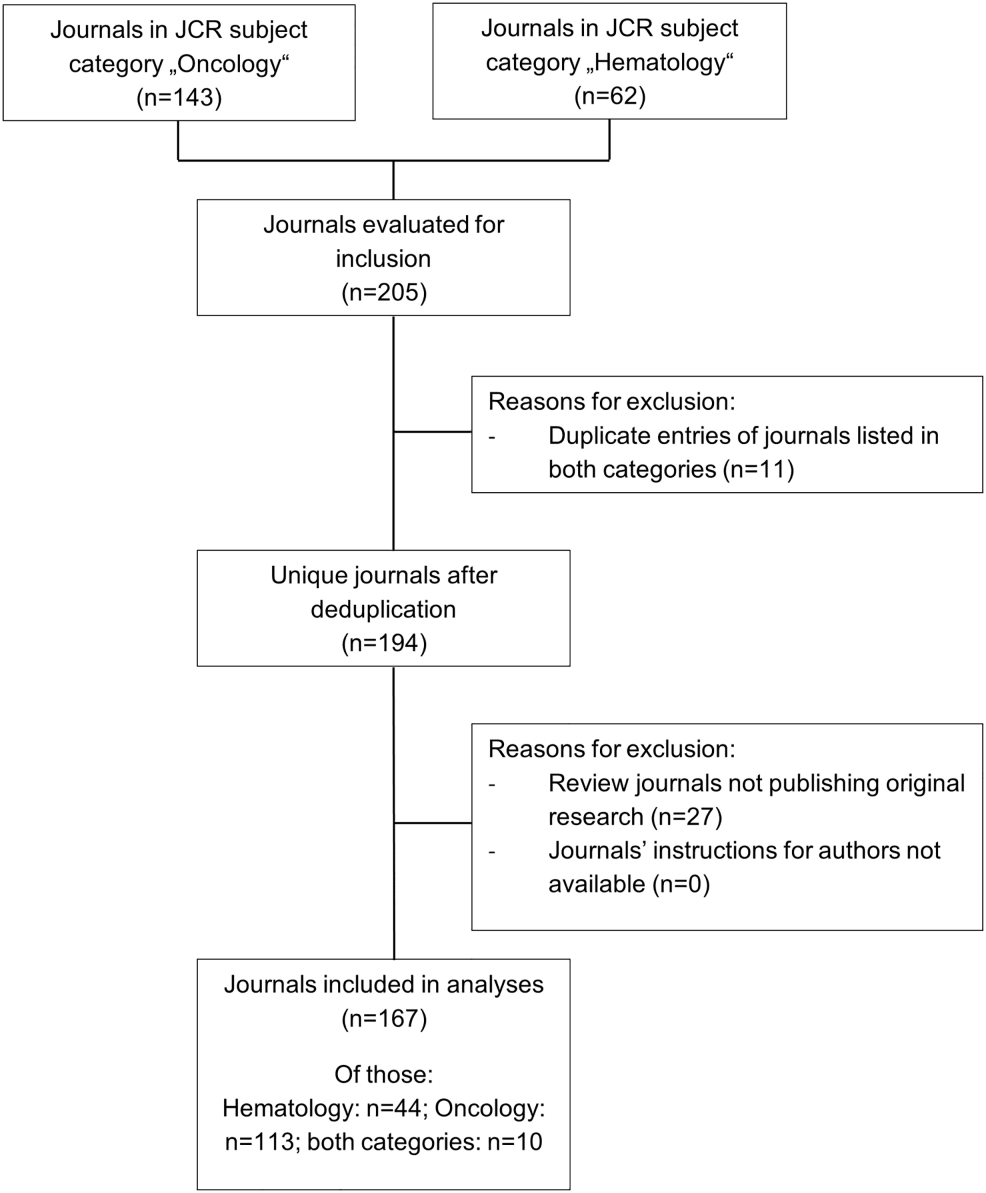
Flow chart—Journal selection for the longitudinal study part.

The study sample for the longitudinal study part consisted of journals listed in the Journal Citation Report 2008 (Science Editions) in the subject categories “Hematology”and “Oncology”(see Fig 2). This was the most recent Journal Citation Report when the study was started in early 2010. Duplicate entries were removed and journals which did not report on original research or whose author instructions were not available online were excluded.

### Variables

For the cross-sectional study part we downloaded the current author instructions and related documents from the journals’ web sites between July and August 2015. These include documents titled: instructions to authors, information for authors, author guides and guidelines, information to contributors, submission guidelines, information of conflicts of interest, journal policies, manuscript guidelines, instructions for manuscript preparation etc. One researcher first read all documents and then used relevant electronic keywords to search the text. The following keywords were used to search the document text for relevant information: Equator, enhanced, consort, consolidated, strobe, stard, moose, quorum, prisma, 1991, 1997, Moher, meta, reporting, ICMJE, international, uniform, committee, regis*, trial, depos*, interest, confl*, comp*. Texts were searched for passages on the following four domains for which we assessed endorsement in the author instructions:

- Endorsement of the ICMJE recommendations
- Endorsement of the following five reporting guidelines
  - Consolidated Standards of Reporting Trials (CONSORT) statement [34, 35]
  - Meta-analysis of Observational Studies in Epidemiology (MOOSE) [36]
  - Preferred Reporting Items for Systematic Reviews and Meta-Analyses (PRISMA) statement (replaced the earlier QUOROM statement) [37, 38]
  - Standards for Reporting of Diagnostic Accuracy (STARD) statement [39–41]
  - Strengthening the Reporting of Observational Studies in Epidemiology (STROBE) statement [42, 43]
  - Mention of the EQUATOR network
- Recommendation/Requirement for trial registration
- Editorial policy for disclosure of conflicts of interests of authors

We define endorsement of any domain as the author instructions saying that they approve or support the use of any of the recommendations. This was done by checking whether the author instructions mentioned the domain or a closely related aspect, e.g., a reference to a publication of a reporting guideline, in the author instructions.

Data were extracted independently in a random sample of 10% of the retrieved journals by a second researcher. Discrepancies were noted in only 2.3% of the entries corresponding to an almost perfect agreement (κ = 1.00 (p>0.001)) [44].

Based on the wording of the author instructions, we determined whether (i) a journal required adherence to a specific domain, for example, by stating that trial registration is a requirement for publication and wording that implies that the paper would not be published without trial registration, (ii) recommended its use, for example, by stating that authors should/ or are recommended to pay attention to a certain reporting guideline, or (iii) did not mention it at all. For conflict of interest statements we distinguished between journals that (i) did not mention whether authors were asked to disclose conflicts of interest, (ii) “would (likely)” publish a statement with the article or whether it (iii) “would (likely) not” publish a statement with the article. Journal characteristics were extracted from the Journal Citation Report or, in case of unavailability, from the National Library of Medicine Catalog of journals referenced in the National Center for Biotechnology Information Databases (http://www.ncbi.nlm.nih.gov/nlmcatalog/journals). Information extracted included start year, language and geographical region of publication, publisher, and journal impact factor.

For the longitudinal study part, author instructions were retrieved from the journals’ websites during three time periods i.e. from March to May 2010, in July 2012 and from July to August 2015, respectively. For follow-up data extraction, we aimed at retrieving data early after the annual impact factor release to enable the timely analyses. In 2010 and 2012 two investigators independently first read the author instructions and then used the following keywords to search the text for passages on the four domains of interest: Equator, enhanced, consort, consolidated, strobe, stard, moose, quorum, prisma, 1991, 1997, Moher, meta, reporting, ICMJE, international, uniform, committee, regis*, trial, depos*, interest, confl*, comp*, disclo*. Any discrepancies were resolved by rechecking the respective web sites and discussion amongst the authors.

All variables were selected prior to conducting the analyses. In the supporting information to this manuscript (S1 File. Data Variables) there is a comprehensive overview of the variables and their values used for the respective models as outlined in the statistical analysis section. We note here that we dichotomized geographical region for use in the models. Using original categories for region of publication caused model convergence problems as there were too few journals from Australasia. So we decided to dichotomize the variable distinguishing journals from North America from those elsewhere, as this resulted in almost equally sized groups (45% vs. 55%) and it also seemed reasonable from a geographical point of view.

### Statistical analysis

We employed a global generalized linear mixed effects model[45], encompassing all domains and all journals by a publisher, to investigate whether there are general effects resulting, e.g., from a general policy (affecting several domains) used for all journals of a publisher (cross-sectional study part). In this global model, publisher-specific effects are modeled by a random intercept, corresponding to different levels of endorsement per publisher. The random intercept takes into account that there are several journals per publisher, and several domains assessed per journal. A second random intercept term is used for the domains, reflecting a potential different level of endorsement per domain. To allow for domain-specific effects of the other covariates (journal impact factor, geographical region, language, start year, and medical category), we fitted one mixed effects model per domain. For journal impact factor we used the log-transformed value as unit steps. Goodness of model fit was achieved using the Akaike Information Criterion (AIC), which selects the actual parameters required for the particular degree of fit by imposing a penalty for increasing the number of parameters. Model quality was measured by considering the log likelihood value from the full model (all parameters included) and the final model (after model selection).

Differences in the trend for endorsement of any domain over time (since 2010) and corresponding variations between the domains (longitudinal study part) were estimated using a generalized linear mixed-effects model with endorsement as response, and a random intercept for the journals to take repeated measurements (sample years 2010, 2012, and 2015) into account. To assess whether trends in endorsement over time differ between domains, we added a random time slope, allowing for different trends for different domains.

To assess whether endorsement of any domain is associated with an increase in the journal impact factor (longitudinal study part) we fitted a linear mixed-effects model with difference in journal impact factor between 2010 and 2014 as outcome and domain endorsement in 2010 as well as further 2010 journal characteristics, such as the publication language (English vs. multi-language), geographical region of publication (not North America vs. North America), as independent variables allowing for random variability between the different domain characteristics. All statistical analyses were conducted using the software environment for statistical computing R, version 3.1.2 [46]. For all mixed models with binary outcome (endorsement yes/no), we used function “glmer” from R package lme4. For the longitudinal analysis investigating potential effects on change in impact factor (continuous outcome), we used function “lmer” from R package lme4. Statistical significance was set at p-value < 0.05.

All original data are available in the supporting information files (S2 File Extracted Data 2010, S3 File Extracted Data 2012, S4 File Extracted Data 2015).

## Results

### Cross-sectional study part

The sample for the cross-sectional part of our study included 231 journals in total (Fig 1).

The sample characteristics are shown in Table 1. The journals were published by 56 publishers; the median number of journals per publisher was 4 (range 1–34).

**Table 1:** Characteristics of the study sample for the cross-sectional study part.

Journal characteristics	Number of journals (%) 2015 (n = 231)
JCR 2014 subject category	
Oncology	172 (74.5)
Hematology	48 (20.8)
Both	11 (4.7)
Publication language	
English	213 (92.2)
Multi-language*	18 (7.8)
Geographical region of publication	
North America	98 (42.4)
UK	49 (21.2)
Australasia	25 (10.8)
Europe without UK	59 (25.6)
Median start year (range)	1992 (1903–2014)
Median impact factor 2014 (range)	2.723 (0.019–24.690)

JCR = Journal Citation Report

*Journals with publications in two or more languages

#### Endorsement of domains in 2015

As of July 2015, the ICMJE recommendations were referred to by 156 journals (67.5%), of which 141 (90.4%) also gave the current Web URL of the ICMJE for further reference (Table 2). The ICMJ recommendations were mentioned in various contexts within the author instructions: some of the journals referred to it as a document for general guidance, others for one or more selected aspects such as ethical considerations (89 journals; 57.0%), manuscript preparation (46 journals; 29.5%) or publication considerations (22 journals; 14.1%).

**Table 2:** Endorsement of domains in author instructions in 2015.

Domains in author instructions	Number of journals (%) 2015 (n = 231)
ICMJE recommendations mentioned	156 (67.5)
ICMJE Web address given	141 (90.4)
CONSORT mentioned	77 (33.3)
CONSORT required	24 (31.2)
CONSORT recommended	53 (68.8)
CONSORT checklist mentioned	28 (36.4)
CONSORT flowchart	25 (32.5)
CONSORT Web URL given	63 (81.8)
CONSORT reference	7 (9.1)
CONSORT explanatory paper mentioned	6 (7.8)
CONSORT extensions mentioned	16 (20.8)
STROBE mentioned	31 (13.4)
STROBE required	11 (35.5)
STROBE recommended	20 (64.5)
STROBE Web URL given	25 (80.7)
STROBE reference	2 (6.5)
STROBE explanatory paper mentioned	1 (3.2)
STARD mentioned	29 (12.6)
STARD required	7 (24.1)
STARD recommended	22 (75.9)
STARD Web URL given	23 (79.3)
STARD reference	3 (10.3)
STARD explanatory paper mentioned	1 (3.5)
PRISMA mentioned	33 (14.3)
PRISMA required	7 (21.2)
PRISMA recommended	26 (78.8)
PRISMA Web URL given	28 (84.8)
PRISMA reference	0 (0.0)
PRISMA explanatory paper mentioned	0 (0.0)
MOOSE mentioned	11 (4.8)
MOOSE required	4 (36.4)
MOOSE recommended	7 (63.6)
MOOSE Web URL given	6 (54.5)
MOOSE reference	1 (9.1)
EQUATOR network mentioned	18 (7.8)
Trial registration mentioned	99 (42.9)
Trial registration required	82 (82.8)
Trial registration recommended	17 (17.2)
Disclosure of CoI mentioned	211 (91.3)
Journal is likely to publish CoI statement	146 (69.2)
Journal is not likely to publish CoI statement	65 (30.8)

CoI = Conflict of interest

The proportions of journals endorsing different reporting guidelines are listed in Table 2.

Trial registration was mentioned by 99 journals (42.9%). Of these, 82 journals (82.8%) required registration of a trial as a precondition for publication and 61 journals (61.6%) also indicated a suitable trial registry such as the ones listed in the WHO International Clinical Trials Registry Platform (http://www.who.int/ictrp/en/), ClinicalTrials.gov (www.clinicaltrials.gov) by the U.S. National Institutes of Health and others.

According to their website, most journals (211; 91.3%) had a policy for disclosure of conflicts of interests of authors. We judged based on provided information in the author instructions that 146 (63.2% of the journals with conflict of interest policy) would publish or consider publishing a conflict of interest statement together with a manuscript.

#### Association of journal characteristics with endorsement in 2015

In the global mixed model log journal impact factor had a strong significant association with general endorsement of any of the domains (OR 1.79 95% confidence interval 1.38 to 2.33). We fitted separate multivariable mixed effects models. For these models, after backward elimination of variables, log journal impact factor mostly had a positive, statistical significant association with endorsement of any of the four domains of interest, except for disclosure of conflicts of interest (Table 3, columns indicated as “Final”). For instance, a change in the log impact factor of 1 was associated with a 1.88-times (95% confidence interval: 1.02 to 3.46) higher odds of endorsement of the ICMJE recommendations. Similar positive associations were found of journal impact factor with endorsement of any of the reporting guidelines and trial registration (Table 3). In addition, geographical region of publication was associated with the endorsement of any domain and trial registration. Journals’ start year was also associated with the endorsement of any domain, but only borderline statistically significant with the endorsement of any reporting guideline. Language of publication and journal subject category did not show significant associations with endorsement of any domain.

**Table 3:** Association of journal characteristics with domain endorsement in 2015 and random effects accounting for publisher or domain-specific variation, type and model quality^§^, for both the full model (Full) containing all covariates as well as the final model (Final) containing AIS-selected variables only.

	Any domain in author instructions		Domains in author instructions							
			ICMJE recommendations		Any reporting guideline		Trial registration		Disclosure of CoI	
	Full	Final	Full	Final	Full	Final	Full	Final	Full	Final
**Fixed effects (OR [95% CI])**										
Change of 1 in log journal impact factor	1.79 [1.38; 2.33]	1.85 [1.43; 2.38]	1.74 [0.91; 3.34] ^Ɨ^	1.88 [1.02; 3.46]	2.62 [1.67; 4.10]	2.58 [1.65 4.03]	2.05 [1.12; 3.78]	2.02 [1.11; 3.64]	3.11 [1.00, 9.69]	2.24 [0.83, 6.02] ^Ɨ^
Not North America* vs. North America	1.98 [1.36; 2.88]	1.97 [1.36; 2.87]	1.89 [0.81 4.42] ^Ɨ^	1.83 [0.80; 4.20] ^Ɨ^	1.66 [0.95; 2.89] ^Ɨ^	1.66 [0.95 2.90] ^Ɨ^	3.85 [1.75; 8.45]	3.85 [1.76; 8.42]	5.51 [1.06, 28.59]	4.47 [0.96, 20.88] ^Ɨ^
Start year (cont.)	1.00 [1.00; 1.01]	1.00 [1.00; 1.01]	1.00 [0.98; 1.02]	-	1.01 [0.99; 1.02] ^Ɨ^	1.01 [0.99 1.02] ^Ɨ^	1.00 [0.99; 1.02]	-	1.02 [0.99, 1.06]	-
English vs. Not English	0.76 [0.37; 1.56]	-	0.50 [0.11; 2.29]	-	1.34 [0.46; 3.88]	-	0.22 [0.03; 1.45] ^Ɨ^	0.24 [0.04; 1.39] ^Ɨ^	3.23 [0.17, 62.14]	-
Oncology vs. hematology	0.12 [0.76; 1.66]	-	0.82 [0.34; 1.97]	-	1.72 [0.88; 3.35] ^Ɨ^	1.73 [0.89 3.37] ^Ɨ^	0.75 [0.33; 1.72]	-	0.76 [0.19, 3.07]	-
**Random intercept effects (variance [SD)]**										
Domain-type	5.331 [2.309]	5.314 [2.305]	n.a.	n.a.	1.291 [1.136]	1.291 [1.136]	n.a.	n.a.	n.a.	n.a.
Publisher	4.172 [2.042]	4.086 [2.021]	3.363 [1.834]	3.346 [1.829]	10.135 [3.184]	10.349 [3.2178]	2.821 [1.68]	2.932 [1.712]	38.34 [6.129]	44.24 [6.651]
**Model quality**										
AIC	1351.7	1348.6	246.1	241.5	685.2	683.5	266.9	264.0	124.7	120.9
Log likelihood	-667.9	-668.3	-116.1	-116.7	-334.6	-334.8	-126.5	-127.0	-55.4	-56.5

CoI = Conflict of Interest; n.a. = not applicable

* Europe plus Australasia plus UK

^§^ Variables not included in the final model are indicated by a dash symbol

^Ɨ^ Variables with borderline statistical significance

Furthermore, there seems to be a strong random intercept effect of publisher on the endorsement of any domain and of the individual domains, especially reporting guidelines (variance > 10) (Table 3).

### Longitudinal study part

The sample of journals used for evaluation of time trends in the longitudinal part of the study is described in Table 4 and the process of sample selection is shown in Fig 2.

**Table 4:** Characteristics of the study sample for the longitudinal study part.

Journal characteristics	Number of journals (%) 2010 (n = 167)
JCR subject category	
Oncology	113 (67.7)
Hematology	44 (26.3)
Both	10 (6.0)
Publication language	
English	156 (93.4)
German	1 (0.6)
Russian	1 (0.6)
Multi-language*	9 (5.4)
Geographical region of publication	
North America	83 (49.7)
UK	39 (23.4)
Australasia	5 (3.0)
Europe without UK	40 (23.9)
Median start year (range)	1986 (1903–2006)
Median impact factor 2008 (range)	2.449 (0.107–24.962)
Median impact factor 2010 (range)	2.772 (0.001–26.925)
Median impact factor 2014 (range)	2.916 (0.101–24.690)

JCR = Journal Citation Report

*Journals with publications in two or more languages

Four journals were not followed up until 2015, because for three of them the author instructions were not found freely available online for analysis in 2015 and one journal was not included in the Journal Citation Report 2014.

#### Trend for endorsement over time (2010–2015)

**Fig 3:**
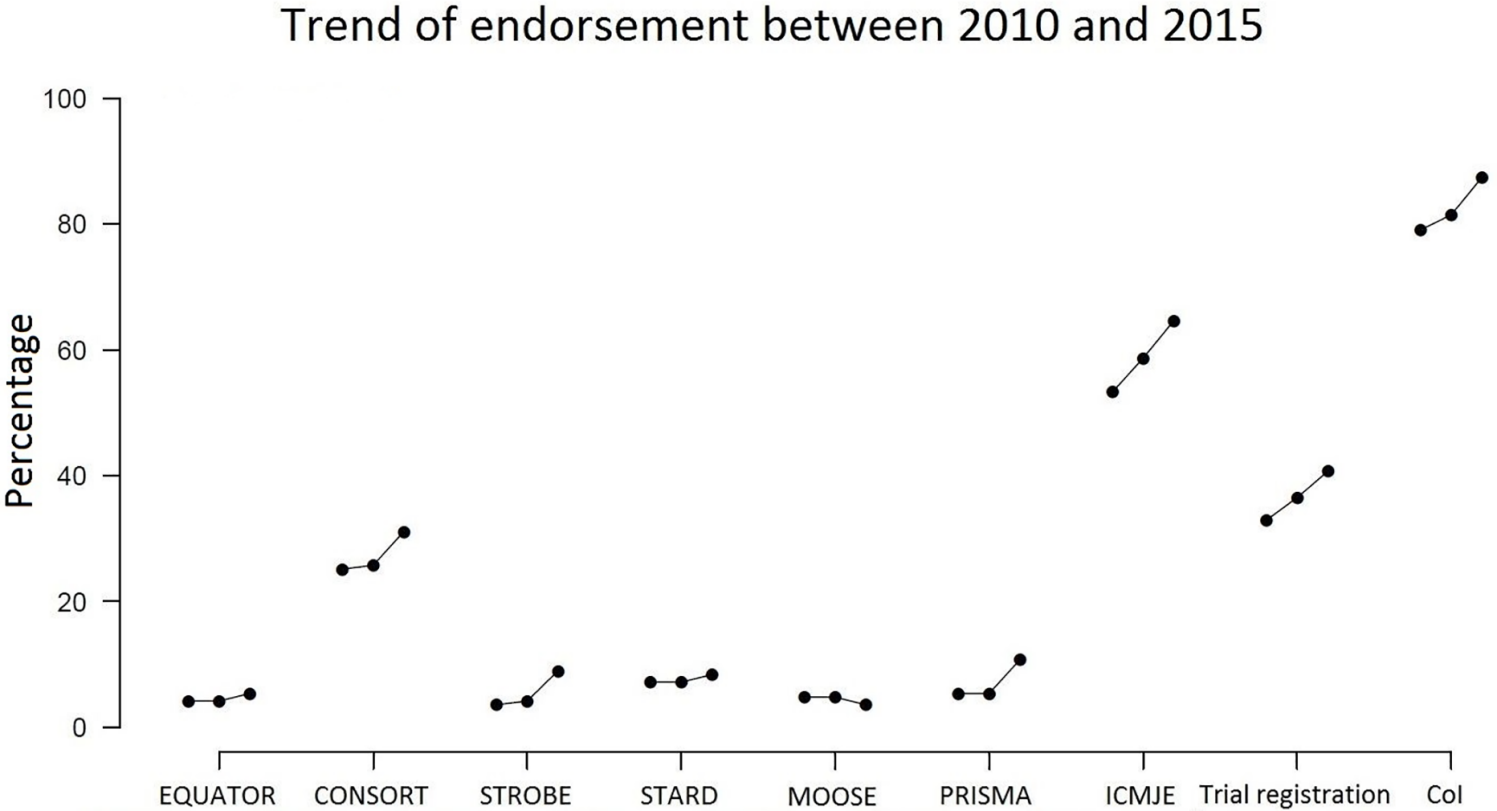
Trend of endorsement between 2010 and 2015. ICMJE = recommendations by the ICMJE; CoI = Conflict of interest. Within each curve the dots show the percentage of journals which endorsed the domains in 2010, 2012 and 2015 respectively (from left to right)

The trend for endorsement of the domains is illustrated in Fig 3, with corresponding numbers and percentage of journals endorsing for each domain given in Table 5.

**Table 5:** Trend of endorsement 2010, 2012 and 2015.

Endorsement of domains in author instructions	Number of journals 2010 (%) (n = 167)	Number of journals 2012 (%) (n = 167)	Number of journals 2015 (%) (n = 163*)
ICMJE recommendations	89 (53.3)	98 (58.7)	108 (66.3)
CONSORT	42 (25.1)	43 (25.8)	52 (31.9)
STROBE	6 (3.6)	7 (4.2)	15 (9.2)
STARD	12 (7.2)	12 (7.2)	14 (8.6)
PRISMA	9 (5.4)	9 (5.4)	18 (11.0)
MOOSE	8 (4.8)	8 (4.8)	6 (3.7)
EQUATOR	7 (4.2)	7 (4.2)	9 (5.5)
Trial registration	55 (32.9)	61 (36.5)	68 (41.7)
Disclosure of conflict of interest	132 (79.0)	136 (81.4)	146 (89.6)

*In the analysis for 2015, three journals had no author instructions available for analysis and one was not included in the Journal Citation Report 2014

Endorsement of any of the four domains increased over time, and this increase was statistically significant in the generalized linear mixed effects model (p≤1e-05, based on asymptotic Wald test for generalized linear mixed-effects model). However, judged from the estimated variance of the random time slope, the magnitude of this increase in endorsement over time did not differ significantly between the different domains (variance < 0.0004).

#### Impact of endorsement in 2010 on journal impact factor difference (2014–2010)

Data suggests that endorsement of any one domain in journals’ author instructions might be positively associated with an individual increase in journal impact factor (RR 1.34, 95% confidence interval -0.95 to 1.89). However, we cannot rule out that this result is due to chance. The type of domain endorsed in the author instructions was not associated with the journal impact factor (random effect variance 0.00). Further journal characteristic associated with a change in journal impact factor were found to be a higher log impact factor in 2010, North America as region of publication and English as publication language (see Table 6).

**Table 6:** Association of journal characteristics with change in journal impact factor (2014–2010) for both the full model (Full) containing all covariates as well as the final model (Final) as a result of model selection.

	Change of 1 in difference of journal impact factor (2014–2010)	
	Full	Final
**Fixed covariate effects (RR [95% CI])**		
Intercept	1.13 [0.47; 2.68]	1.48 [0.79; 2.77]
Endorsement of any domain	1.34 [0.95; 1.88]	1.34 [0.95; 1.89]
Log impact factor 2010	0.53 [0.45; 0.62]	0.53 [0.45; 0.62]
Not North America vs. North America	0.54 [0.40; 0.74]	0.52 [0.39; 0.71]
Start year (cont.)	1.00 [0.99; 1.01]	-
English vs. Not English	1.81 [0.97; 3.38]	1.86 [1.01; 3.45]
Oncology vs. hematology	1.18 [0.86; 1.62]	-
**Random effects (variance [SD])**		
Domain-type	0.000 [0.000]	0.000 [0.000]
**Model quality**		
Log likelihood	-3194.3	-3189.5

## Discussion

We analyzed to which extent author instructions of hematology and oncology journals provide guidance on different aspects of reporting quality and good publication practice. The ICMJE recommendations were mentioned by about two thirds of journals. The most commonly endorsed reporting guideline was the CONSORT statement, but it was referred to by only about a third of journals. Trial registration was mentioned by about 40% of the journals of which the majority required it as precondition for publication. In journals of hematology and oncology conflict of interest policies were laid out most often as was also shown recently by Kesselheim et al. [47]. Of several journal characteristics, the journal impact factor consistently showed a significant positive association with endorsement of any reporting guideline, the recommendations by the ICMJE and trial registration while start year and geographical region of journals were associated with endorsement of fewer of the domains in the author instructions. Despite the intrinsic weaknesses of the journal impact factor as a quality indicator [48], our study confirms its association with good publication practice as found in previous studies [11, 19, 25, 49]. Our study has shown that the endorsement of any of the domains is likely also associated with a stronger increase in journal impact factor.

We found the highest proportion of endorsement for a policy on disclosure of conflict of interest, low proportions who mentioned the registration of clinical trials and the lowest proportion of endorsement for reporting guidelines. Interestingly, this pattern seems to be very consistent across different medical fields including general medicine [14, 50], psychiatry [21], pediatrics [19, 20], surgery [24, 25] and urology [51, 52]. For instance, trial registration was required or recommended by 23% of pediatric journals indexed in the Journal Citation Report [19] compared to 35.5% of hematology and oncology journals. It is important to note that, although one third of hematology and oncology journals require or recommend trial registration, nearly two thirds still do not mention trial registration in their author instructions. It has to be noted that our sample included journals that do not publish research in humans but only studies for which trial registration is rarely applicable, such as basic research. However, the description of the scope and content of a journal as well as its author instructions and the tables of content are often not distinct enough to distinguish journals that publish human studies and those that do not. Therefore it is challenging to correctly determine which journals’ author instructions should actually endorse trial registration.

The consistency of the inclusion of the different domains across different subsets of journals suggests that journal editors consider disclosure of conflict of interest to be more important than registration of clinical trials or use of reporting guidelines. This lack of attention to the latter two issues is problematic given the importance of transparent and complete reporting for the translation of published study results into clinical practice [8, 53]. This is astounding given that empirical data have shown that only about 10% of 262 randomized controlled trials in oncology completely reported on a set of 10 items deemed necessary to fully describe the applied therapeutic intervention and thereby allow for replication of the trial intervention [2]. Other studies used scores based on the CONSORT statement and found moderate or poor reporting quality of oncology trials but some improvement over time e.g. in a set of 72 sarcoma trials published between 1988 and 2008 [54]. The overall reporting quality was also poor in 44 palliative oncology trials published between 2004 and 2009 [55].

The strengths of our study include a comprehensive and transparent selection of high profile journals as study sample as well as a very comprehensive list of items investigated compared to previous studies about author instructions for hematology and oncology journals [32]. Furthermore, unlike most other studies of author instructions, we looked not only at the status quo but analyzed also the change over time. High standards for the retrieval and extraction of the data were used. Besides the author instructions other available documents, for example, ethical guidelines of the journal were retrieved and searched. The data extraction was conducted manually with the help of text word searches and reading each document carefully and reached nearly perfect agreement in a 10% sample. Lastly, the statistical analyses were thorough and considered confounding factors which might have influenced the results.

There are some limitations to our study. First, since the Journal Citation Report covers only a selection of hematology and oncology journals the study sample might not be representative of all journals in this field and in particular not of non-English journals. Second, we did not investigate whether and how author instructions were implemented and endorsed in practice or whether other policies that might impact on the editorial process, such as peer review policies were in place. It also might well be that various aspects within author instructions are not necessarily enforced in everyday editorial practice [56]. Our study reviewed potentially volatile online-resources, which are subject to ongoing change and updates. Hence, information in the author instructions might have changed in the meantime. Lastly, we have only analyzed author instructions which were publicly accessible online. Some journals might provide more detailed author instructions after requiring authors to log into a platform or managing software. Therefore, we might have missed information which is only available during the actual submission process.

In order to increase the uptake of measures that aim at improving reporting quality and making research transparent and reproducible it is recommended that editors endorse them in author instructions [57] and make them accessible to authors [58]. Consequently editorial teams and peer-reviewers should pay close attention to the adherence to transparency and reporting guidelines by authors [57]. The editorial process could be enhanced by making it a two-step process where editors or peer-reviewers first check for the endorsement of relevant recommendations for increasing reporting quality and then review the content of the manuscript. The editorial process could also be supported by semi-automated checking for the endorsement of recommendations.

In conclusion, major hematology and oncology journals have not yet implemented the four domains aiming to improve publication practice in their author instructions as widely as would be needed to improve the published record of cancer research. The promotion of reporting guidelines and enforcement of generally accepted standards, such as prospective trial registration, could further improve the utility of published study results. Transparency is a sine qua non for clinical research that ultimately aims to translate into better quality of care and health for patients.

## Supporting information

S1 File Data variables. (DOCX)

S2 File Extracted data 2010. (XLS)

S3 File Extracted data 2012. (XLS)

S4 File Extracted data 2015. (XLS)
