# Supplementary material for: Guidance in author instructions of hematology and oncology journals: A cross sectional and longitudinal study
Source: PLoS One. 2017 Apr 28;12(4):e0176489. doi: 10.1371/journal.pone.0176489 (PMC5409080; doi:10.1371/journal.pone.0176489)
Supplement: S1 File — (DOCX) [file pone.0176489.s001.docx]

| Variable name | Variable label | Variable description | Allowable values | Variable type | Measurement scale | Variable analysed as .. |
| --- | --- | --- | --- | --- | --- | --- |
| ICMJE | ICMJE recommendations | Was there any mention of the ICMJE recommendations. | Yes = mentioned  No = not mentioned | Text | Categorical | Dependent for cross sectional study part; independent for longitudinal study part |
| EQUATOR | EQUATOR | Was EQUATOR (i) mentioned or referred to or (ii) not mentioned. | Yes = mentioned  No = not mentioned | Text | Categorical | Descriptive only |
| ‘Guides’ or ‘Guidelines’ | Reporting guidelines overall | Was any reporting guideline (i) mentioned or (ii) not mentioned. | Yes = mentioned  No = not mentioned | Text | Categorical | Dependent for cross sectional study part; independent for longitudinal study part |
| CONSORT | CONSORT | Was CONSORT (i) mentioned or referred to or (ii) not mentioned. | Yes = mentioned  No = not mentioned | Text | Categorical | Dependent for cross sectional study part; independent for longitudinal study part |
| CON_RERE | CONSORT recommended or required | Did the wording in the author instructions imply that the reporting guideline was (i) recommended for use or (ii) required, implying that the manuscript might not be considered for publication when the reporting guideline was not adhered to. | Req = required  Rec = recommended | Text | Categorical | Descriptive only |
| STROBE | STROBE | Was STROBE (i) mentioned or referred to or (ii) not mentioned. | Yes = mentioned  No = not mentioned | Text | Categorical | Dependent for cross sectional study part; independent for longitudinal study part |
| STR_RERE | STROBE recommended or required | Did the wording in the author instructions imply that the reporting guideline was (i) recommended for use or (ii) required, implying that the manuscript might not be considered for publication when the reporting guideline was not adhered to. | Req = required  Rec = recommended | Text | Categorical | Descriptive only |
| STARD | STARD | Was STARD (i) mentioned or referred to or (ii) not mentioned. | Yes = mentioned  No = not mentioned | Text | Categorical | Dependent for cross sectional study part; independent for longitudinal study part |
| STA_RERE | STARD recommended or required | Did the wording in the author instructions imply that the reporting guideline was (i) recommended for use or (ii) required, implying that the manuscript might not be considered for publication when the reporting guideline was not adhered to. | Req = required  Rec = recommended | Text | Categorical | Descriptive only |
| MOOSE | MOOSE | Was MOOSE (i) mentioned or referred to or (ii) not mentioned. | Yes = mentioned  No = not mentioned | Text | Categorical | Dependent for cross sectional study part; independent for longitudinal study part |
| MOO_RERE | MOOSE recommended or required | Did the wording in the author instructions imply that the reporting guideline was (i) recommended for use or (ii) required, implying that the manuscript might not be considered for publication when the reporting guideline was not adhered to. | Req = required  Rec = recommended | Text | Categorical | Descriptive only |
| QUORUM | PRISMA | Was PRISMA (i) mentioned or referred to or (ii) not mentioned. | Yes = mentioned  No = not mentioned | Text | Categorical | Dependent for cross sectional study part; independent for longitudinal study part |
| QUO_RERE | PRISMA recommended or required | Did the wording in the author instructions imply that the reporting guideline was (i) recommended for use or (ii) required, implying that the manuscript might not be considered for publication when the reporting guideline was not adhered to. | Req = required  Rec = recommended | Text | Categorical | Descriptive only |
| TRIALREG | Trial registration | Was a recommendation to register trials (i) mentioned or referred to or (ii) not mentioned. | Yes = mentioned  No = not mentioned | Text | Categorical | Dependent for cross sectional study part; independent for longitudinal study part |
| TRI_RERE | Trial registration recommended or required | Did the wording in the author instructions imply that trial registration was (i) recommended or (ii) required, implying that the manuscript might not be considered for publication when the trial was not registered. | Req = required  Rec = recommended | Text | Categorical | Descriptive only |
| COI_AUTH | Conflict of interest declaration | Was a recommendation to declare conflicts of interests (i) mentioned or referred to or (ii) not mentioned. | Yes = mentioned  No = not mentioned | Text | Categorical | Dependent for cross sectional study part; independent for longitudinal study part |
| COI_PUBL | Conflict of interest declaration will be / will not be published or publication of declaration unclear | Did the wording in the author instructions imply that the declaration of conflicts of interest (i) will be published (ii) will not be published or was there (iii) no information regarding the publication of the declaration of conflicts of interest. | Yes = will be published  No = will not be published  Unclear = unclear | Text | Categorical | Dependent for cross sectional study part; independent for longitudinal study part |
| Onc vs. hem | Journal subject category | Was the journal listed in the journal listed in the Journal Citation Report (Science Edition) category (i) “Hematology” or (ii) “Oncology” or (iii) both categories. | Hematology  Oncology  Both | Text | Categorical | Independent for cross sectional study part |
| English vs. not english | Publication language | Was the publication language (i) English or (ii) another or more than one language. | English  Other and multi-language | Text | Categorical | Independent for cross sectional study part |
| Not NorthA vs. NorthA | Geographical region of publication | Was the main editorial base of the journal in (i) Australasia, (ii) Europe, (iii) North America, (iv) UK. | Not North America vs. North America | Text | Categorical | Independent for cross sectional study part |
| start_year | Start year | What was the start year. | Min - max | Number | Continuous | Independent for cross sectional study part |
| IF_2010 / log(IF_2014) | Log impact factor 2010 or 2014 | Log median impact factor | min - max | Number | Continuous | Independent for cross sectional study part |
| diffIF | - | Difference between impact factor 2010 and 2014; IF_2014 – IF_2010 | min – max | Number | Continuous | Dependent for longitudinal study part |
| qualiyes | Endorsement of any domain | Was any domain included in the authors instructions. | 0, 1 | Number | Categorical | Dependent for longitudinal study part |
